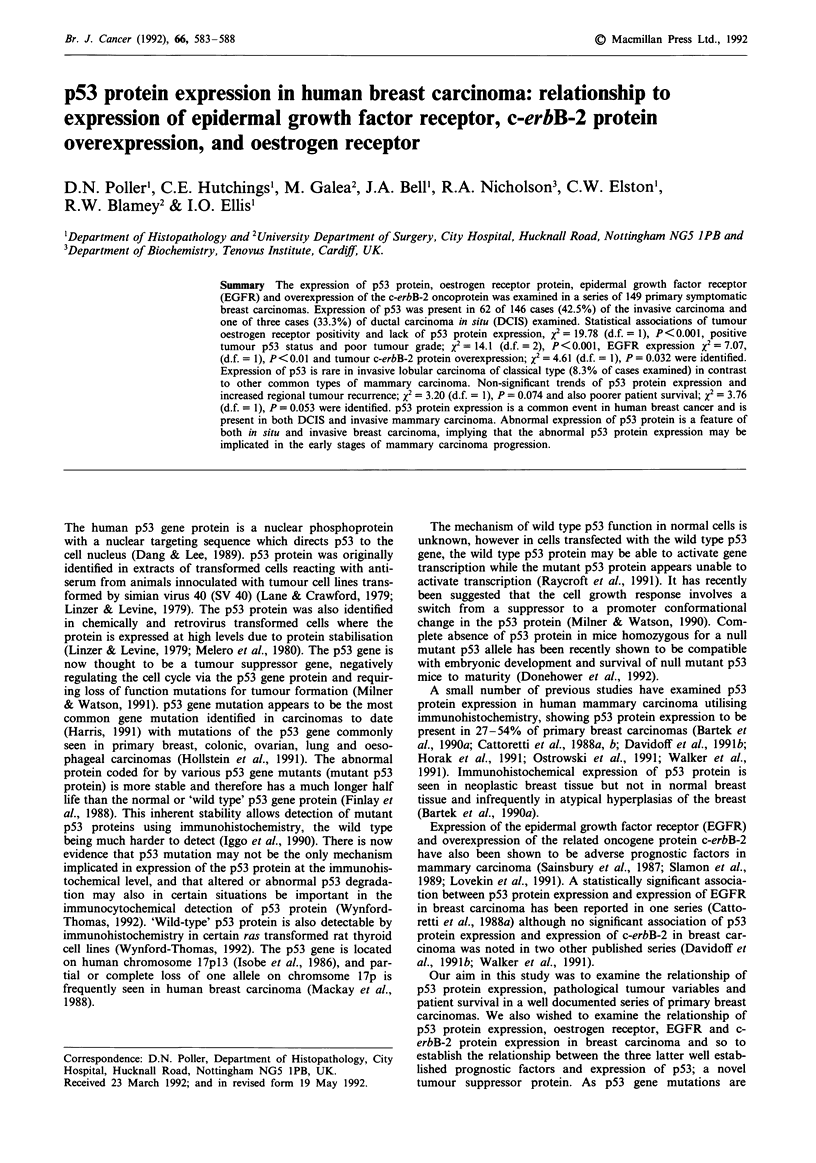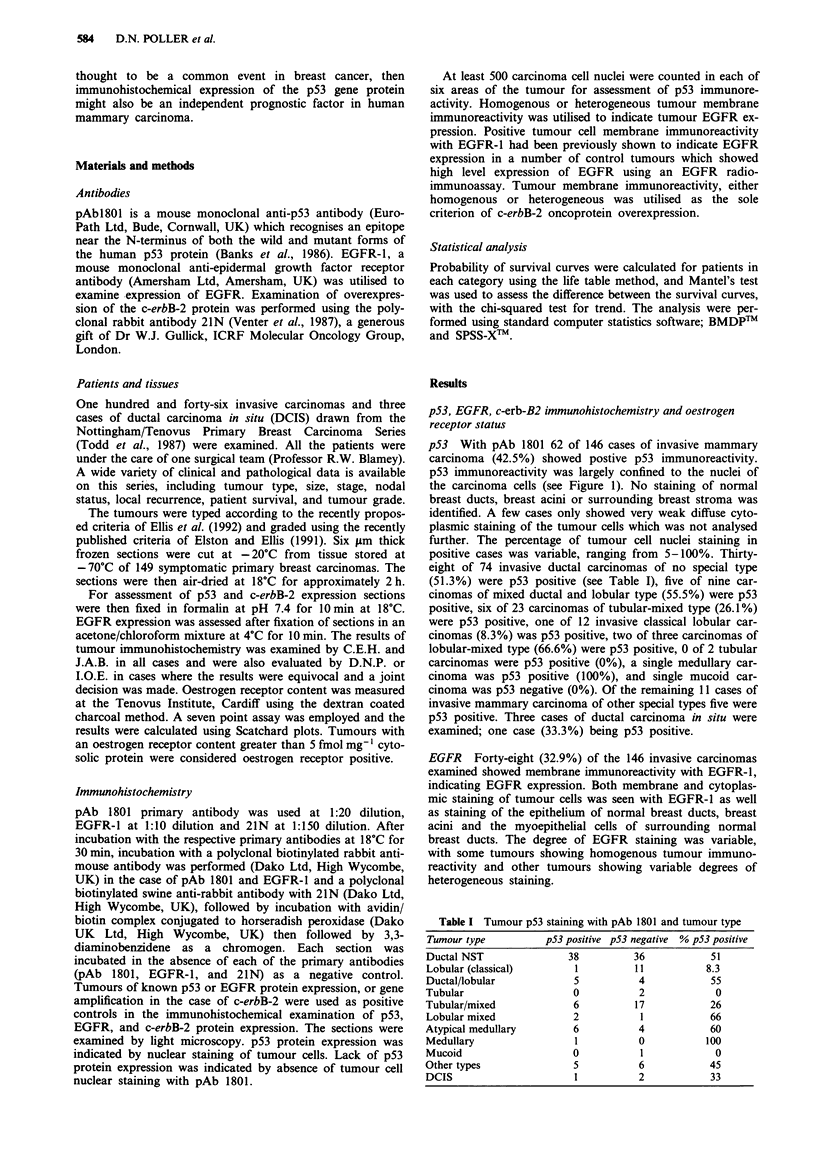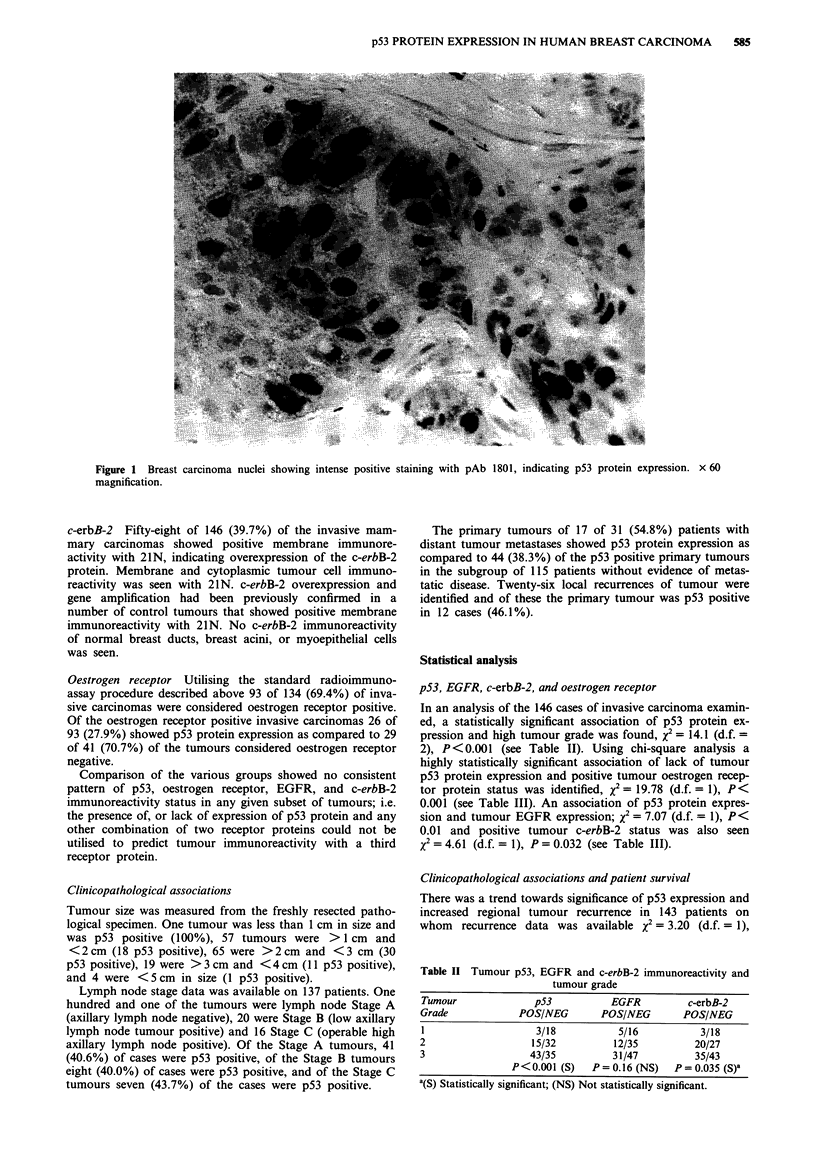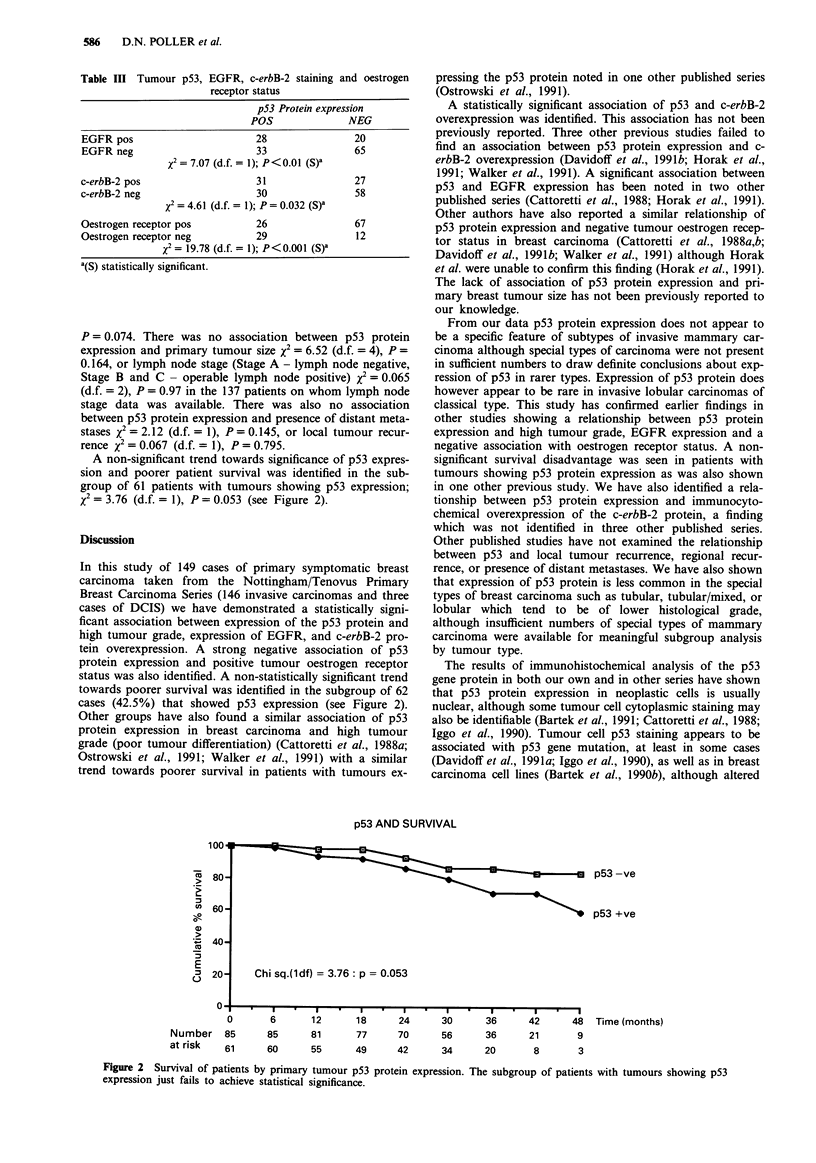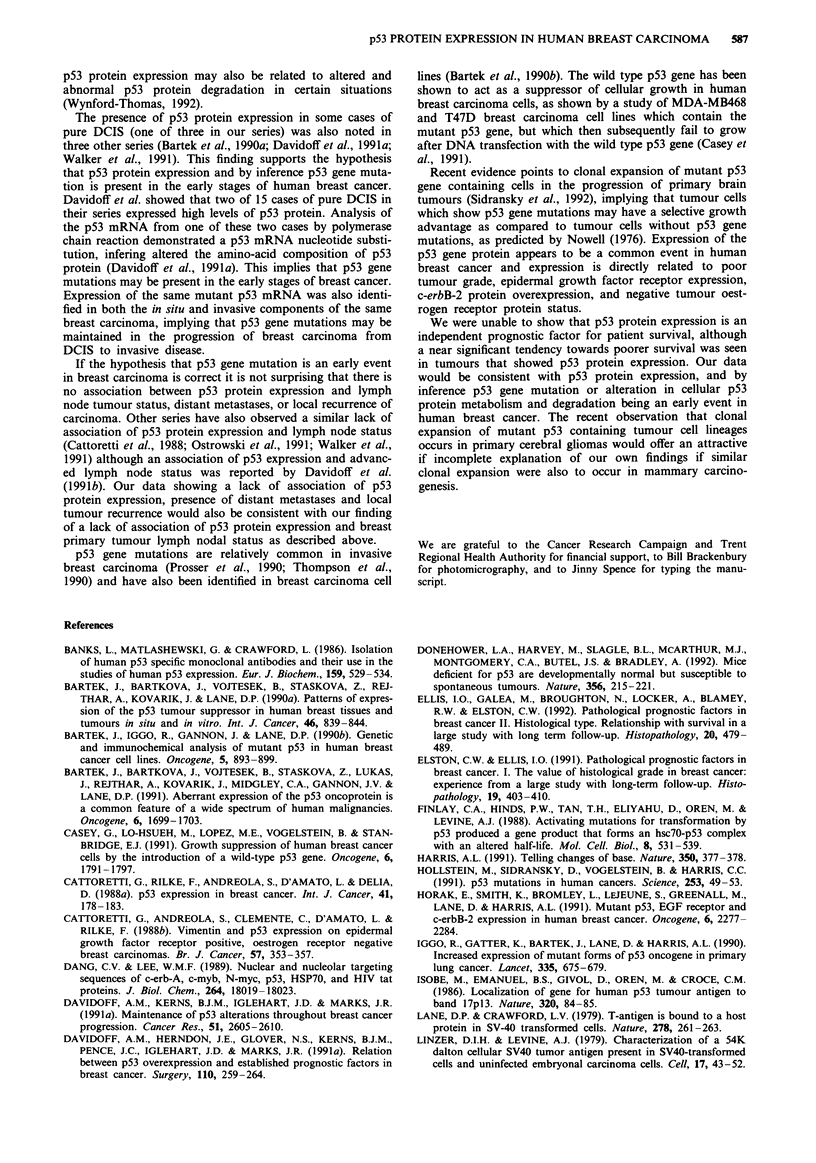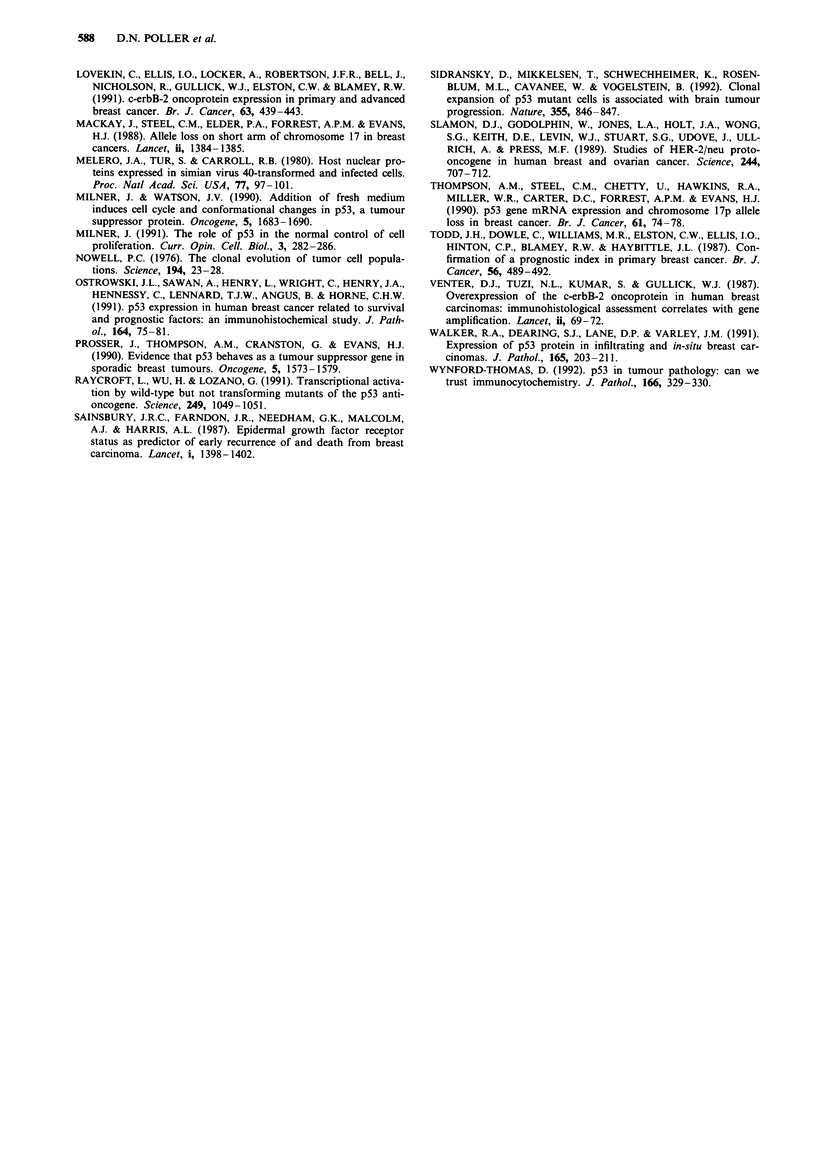# p53 protein expression in human breast carcinoma: relationship to expression of epidermal growth factor receptor, c-erbB-2 protein overexpression, and oestrogen receptor.

**DOI:** 10.1038/bjc.1992.318

**Published:** 1992-09

**Authors:** D. N. Poller, C. E. Hutchings, M. Galea, J. A. Bell, R. A. Nicholson, C. W. Elston, R. W. Blamey, I. O. Ellis

**Affiliations:** Department of Histopathology, City Hospital, Nottingham, UK.

## Abstract

**Images:**


 © Macmillan Press Ltd., 1992

### p53 protein expression in human breast carcinoma: relationship to expression of epidermal growth factor receptor, c-*erb*B-2 protein overexpression, and oestrogen receptor

D.N. Poller[1], C.E. Hutchings[1], M. Galea[2], J.A. Bell[1], R.A. Nicholson[3], C.W. Elston[1], R.W. Blamey[2] & I.O. Ellis[1]

[1]*Department of Histopathology and* [2]*University Department of Surgery, City Hospital, Hucknall Road, Nottingham NG5 1PB and* [3]*Department of Biochemistry, Tenovus Institute, Cardiff, UK.*

**Summary** The expression of p53 protein, oestrogen receptor protein, epidermal growth factor receptor (EGFR) and overexpression of the c-*erb*B-2 oncoprotein was examined in a series of 149 primary symptomatic breast carcinomas. Expression of p53 was present in 62 of 146 cases (42.5%) of the invasive carcinoma and one of three cases (33.3%) of ductal carcinoma *in situ* (DCIS) examined. Statistical associations of tumour oestrogen receptor positivity and lack of p53 protein expression, $\chi^2 = 19.78$ (d.f. = 1), $P<0.001$, positive tumour p53 status and poor tumour grade; $\chi^2 = 14.1$ (d.f. = 2), $P<0.001$, EGFR expression $\chi^2 = 7.07$, (d.f. = 1), $P<0.01$ and tumour c-*erb*B-2 protein overexpression; $\chi^2 = 4.61$ (d.f. = 1), $P = 0.032$ were identified. Expression of p53 is rare in invasive lobular carcinoma of classical type (8.3% of cases examined) in contrast to other common types of mammary carcinoma. Non-significant trends of p53 protein expression and increased regional tumour recurrence; $\chi^2 = 3.20$ (d.f. = 1), $P = 0.074$ and also poorer patient survival; $\chi^2 = 3.76$ (d.f. = 1), $P = 0.053$ were identified. p53 protein expression is a common event in human breast cancer and is present in both DCIS and invasive mammary carcinoma. Abnormal expression of p53 protein is a feature of both *in situ* and invasive breast carcinoma, implying that the abnormal p53 protein expression may be implicated in the early stages of mammary carcinoma progression.

The human p53 gene protein is a nuclear phosphoprotein with a nuclear targeting sequence which directs p53 to the cell nucleus (Dang & Lee, 1989). p53 protein was originally identified in extracts of transformed cells reacting with antiserum from animals innoculated with tumour cell lines transformed by simian virus 40 (SV 40) (Lane & Crawford, 1979; Linzer & Levine, 1979). The p53 protein was also identified in chemically and retrovirus transformed cells where the protein is expressed at high levels due to protein stabilisation (Linzer & Levine, 1979; Melero *et al.*, 1980). The p53 gene is now thought to be a tumour suppressor gene, negatively regulating the cell cycle via the p53 gene protein and requiring loss of function mutations for tumour formation (Milner & Watson, 1991). p53 gene mutation appears to be the most common gene mutation identified in carcinomas to date (Harris, 1991) with mutations of the p53 gene commonly seen in primary breast, colonic, ovarian, lung and oesophageal carcinomas (Hollstein *et al.*, 1991). The abnormal protein coded for by various p53 gene mutants (mutant p53 protein) is more stable and therefore has a much longer half life than the normal or 'wild type' p53 gene protein (Finlay *et al.*, 1988). This inherent stability allows detection of mutant p53 proteins using immunohistochemistry, the wild type being much harder to detect (Iggo *et al.*, 1990). There is now evidence that p53 mutation may not be the only mechanism implicated in expression of the p53 protein at the immunohistochemical level, and that altered or abnormal p53 degradation may also in certain situations be important in the immunocytochemical detection of p53 protein (Wynford-Thomas, 1992). 'Wild-type' p53 protein is also detectable by immunohistochemistry in certain *ras* transformed rat thyroid cell lines (Wynford-Thomas, 1992). The p53 gene is located on human chromosome 17p13 (Isobe *et al.*, 1986), and partial or complete loss of one allele on chromsome 17p is frequently seen in human breast carcinoma (Mackay *et al.*, 1988).

Correspondence: D.N. Poller, Department of Histopathology, City Hospital, Hucknall Road, Nottingham NG5 1PB, UK.
Received 23 March 1992; and in revised form 19 May 1992.

The mechanism of wild type p53 function in normal cells is unknown, however in cells transfected with the wild type p53 gene, the wild type p53 protein may be able to activate gene transcription while the mutant p53 protein appears unable to activate transcription (Raycroft *et al.*, 1991). It has recently been suggested that the cell growth response involves a switch from a suppressor to a promoter conformational change in the p53 protein (Milner & Watson, 1990). Complete absence of p53 protein in mice homozygous for a null mutant p53 allele has been recently shown to be compatible with embryonic development and survival of null mutant p53 mice to maturity (Donehower *et al.*, 1992).

A small number of previous studies have examined p53 protein expression in human mammary carcinoma utilising immunohistochemistry, showing p53 protein expression to be present in 27-54% of primary breast carcinomas (Bartek *et al.*, 1990*a*; Cattoretti *et al.*, 1988*a*, *b*; Davidoff *et al.*, 1991*b*; Horak *et al.*, 1991; Ostrowski *et al.*, 1991; Walker *et al.*, 1991). Immunohistochemical expression of p53 protein is seen in neoplastic breast tissue but not in normal breast tissue and infrequently in atypical hyperplasias of the breast (Bartek *et al.*, 1990*a*).

Expression of the epidermal growth factor receptor (EGFR) and overexpression of the related oncogene protein c-*erb*B-2 have also been shown to be adverse prognostic factors in mammary carcinoma (Sainsbury *et al.*, 1987; Slamon *et al.*, 1989; Lovekin *et al.*, 1991). A statistically significant association between p53 protein expression and expression of EGFR in breast carcinoma has been reported in one series (Cattoretti *et al.*, 1988*a*) although no significant association of p53 protein expression and expression of c-*erb*B-2 in breast carcinoma was noted in two other published series (Davidoff *et al.*, 1991*b*; Walker *et al.*, 1991).

Our aim in this study was to examine the relationship of p53 protein expression, pathological tumour variables and patient survival in a well documented series of primary breast carcinomas. We also wished to examine the relationship of p53 protein expression, oestrogen receptor, EGFR and c-*erb*B-2 protein expression in breast carcinoma and so to establish the relationship between the three latter well established prognostic factors and expression of p53; a novel tumour suppressor protein. As p53 gene mutations are thought to be a common event in breast cancer, then immunohistochemical expression of the p53 gene protein might also be an independent prognostic factor in human mammary carcinoma.

### Materials and methods

### *Antibodies*

pAb1801 is a mouse monoclonal anti-p53 antibody (Euro-Path Ltd, Bude, Cornwall, UK) which recognises an epitope near the N-terminus of both the wild and mutant forms of the human p53 protein (Banks *et al.*, 1986). EGFR-1, a mouse monoclonal anti-epidermal growth factor receptor antibody (Amersham Ltd, Amersham, UK) was utilised to examine expression of EGFR. Examination of overexpression of the c-*erb*B-2 protein was performed using the polyclonal rabbit antibody 21N (Venter *et al.*, 1987), a generous gift of Dr W.J. Gullick, ICRF Molecular Oncology Group, London.

### *Patients and tissues*

One hundred and forty-six invasive carcinomas and three cases of ductal carcinoma *in situ* (DCIS) drawn from the Nottingham/Tenovus Primary Breast Carcinoma Series (Todd *et al.*, 1987) were examined. All the patients were under the care of one surgical team (Professor R.W. Blamey). A wide variety of clinical and pathological data is available on this series, including tumour type, size, stage, nodal status, local recurrence, patient survival, and tumour grade.

The tumours were typed according to the recently proposed criteria of Ellis *et al.* (1992) and graded using the recently published criteria of Elston and Ellis (1991). Six μm thick frozen sections were cut at −20°C from tissue stored at −70°C of 149 symptomatic primary breast carcinomas. The sections were then air-dried at 18°C for approximately 2 h.

For assessment of p53 and c-*erb*B-2 expression sections were then fixed in formalin at pH 7.4 for 10 min at 18°C. EGFR expression was assessed after fixation of sections in an acetone/chloroform mixture at 4°C for 10 min. The results of tumour immunohistochemistry was examined by C.E.H. and J.A.B. in all cases and were also evaluated by D.N.P. or I.O.E. in cases where the results were equivocal and a joint decision was made. Oestrogen receptor content was measured at the Tenovus Institute, Cardiff using the dextran coated charcoal method. A seven point assay was employed and the results were calculated using Scatchard plots. Tumours with an oestrogen receptor content greater than 5 fmol mg$^{-1}$ cytosolic protein were considered oestrogen receptor positive.

### *Immunohistochemistry*

pAb 1801 primary antibody was used at 1:20 dilution, EGFR-1 at 1:10 dilution and 21N at 1:150 dilution. After incubation with the respective primary antibodies at 18°C for 30 min, incubation with a polyclonal biotinylated rabbit anti-mouse antibody was performed (Dako Ltd, High Wycombe, UK) in the case of pAb 1801 and EGFR-1 and a polyclonal biotinylated swine anti-rabbit antibody with 21N (Dako Ltd, High Wycombe, UK), followed by incubation with avidin/biotin complex conjugated to horseradish peroxidase (Dako UK Ltd, High Wycombe, UK) then followed by 3,3-diaminobenzidene as a chromogen. Each section was incubated in the absence of each of the primary antibodies (pAb 1801, EGFR-1, and 21N) as a negative control. Tumours of known p53 or EGFR protein expression, or gene amplification in the case of c-*erb*B-2 were used as positive controls in the immunohistochemical examination of p53, EGFR, and c-*erb*B-2 protein expression. The sections were examined by light microscopy. p53 protein expression was indicated by nuclear staining of tumour cells. Lack of p53 protein expression was indicated by absence of tumour cell nuclear staining with pAb 1801.

At least 500 carcinoma cell nuclei were counted in each of six areas of the tumour for assessment of p53 immunoreactivity. Homogenous or heterogeneous tumour membrane immunoreactivity was utilised to indicate tumour EGFR expression. Positive tumour cell membrane immunoreactivity with EGFR-1 had been previously shown to indicate EGFR expression in a number of control tumours which showed high level expression of EGFR using an EGFR radioimmunoassay. Tumour membrane immunoreactivity, either homogenous or heterogeneous was utilised as the sole criterion of c-*erb*B-2 oncoprotein overexpression.

### *Statistical analysis*

Probability of survival curves were calculated for patients in each category using the life table method, and Mantel's test was used to assess the difference between the survival curves, with the chi-squared test for trend. The analysis were performed using standard computer statistics software; BMDP™ and SPSS-X™.

### Results

### *p53, EGFR, c-*erb*-B2 immunohistochemistry and oestrogen receptor status*

*p53* With pAb 1801 62 of 146 cases of invasive mammary carcinoma (42.5%) showed postive p53 immunoreactivity. p53 immunoreactivity was largely confined to the nuclei of the carcinoma cells (see Figure 1). No staining of normal breast ducts, breast acini or surrounding breast stroma was identified. A few cases only showed very weak diffuse cytoplasmic staining of the tumour cells which was not analysed further. The percentage of tumour cell nuclei staining in positive cases was variable, ranging from 5–100%. Thirty-eight of 74 invasive ductal carcinomas of no special type (51.3%) were p53 positive (see Table I), five of nine carcinomas of mixed ductal and lobular type (55.5%) were p53 positive, six of 23 carcinomas of tubular-mixed type (26.1%) were p53 positive, one of 12 invasive classical lobular carcinomas (8.3%) was p53 positive, two of three carcinomas of lobular-mixed type (66.6%) were p53 positive, 0 of 2 tubular carcinomas were p53 positive (0%), a single medullary carcinoma was p53 positive (100%), and single mucoid carcinoma was p53 negative (0%). Of the remaining 11 cases of invasive mammary carcinoma of other special types five were p53 positive. Three cases of ductal carcinoma *in situ* were examined; one case (33.3%) being p53 positive.

*EGFR* Forty-eight (32.9%) of the 146 invasive carcinomas examined showed membrane immunoreactivity with EGFR-1, indicating EGFR expression. Both membrane and cytoplasmic staining of tumour cells was seen with EGFR-1 as well as staining of the epithelium of normal breast ducts, breast acini and the myoepithelial cells of surrounding normal breast ducts. The degree of EGFR staining was variable, with some tumours showing homogenous tumour immunoreactivity and other tumours showing variable degrees of heterogeneous staining.

**Table I** Tumour p53 staining with pAb 1801 and tumour type

| *Tumour type* | *p53 positive* | *p53 negative* | *% p53 positive* |
|---|---|---|---|
| Ductal NST | 38 | 36 | 51 |
| Lobular (classical) | 1 | 11 | 8.3 |
| Ductal/lobular | 5 | 4 | 55 |
| Tubular | 0 | 2 | 0 |
| Tubular/mixed | 6 | 17 | 26 |
| Lobular mixed | 2 | 1 | 66 |
| Atypical medullary | 6 | 4 | 60 |
| Medullary | 1 | 0 | 100 |
| Mucoid | 0 | 1 | 0 |
| Other types | 5 | 6 | 45 |
| DCIS | 1 | 2 | 33 |

**Figure 1** Breast carcinoma nuclei showing intense positive staining with pAb 1801, indicating p53 protein expression. ×60 magnification.

*c-*erb*B-2* Fifty-eight of 146 (39.7%) of the invasive mammary carcinomas showed positive membrane immunoreactivity with 21N, indicating overexpression of the c-*erb*B-2 protein. Membrane and cytoplasmic tumour cell immunoreactivity was seen with 21N. c-*erb*B-2 overexpression and gene amplification had been previously confirmed in a number of control tumours that showed positive membrane immunoreactivity with 21N. No c-*erb*B-2 immunoreactivity of normal breast ducts, breast acini, or myoepithelial cells was seen.

*Oestrogen receptor* Utilising the standard radioimmunoassay procedure described above 93 of 134 (69.4%) of invasive carcinomas were considered oestrogen receptor positive. Of the oestrogen receptor positive invasive carcinomas 26 of 93 (27.9%) showed p53 protein expression as compared to 29 of 41 (70.7%) of the tumours considered oestrogen receptor negative.

Comparison of the various groups showed no consistent pattern of p53, oestrogen receptor, EGFR, and c-*erb*B-2 immunoreactivity status in any given subset of tumours; i.e. the presence of, or lack of expression of p53 protein and any other combination of two receptor proteins could not be utilised to predict tumour immunoreactivity with a third receptor protein.

### *Clinicopathological associations*

Tumour size was measured from the freshly resected pathological specimen. One tumour was less than 1 cm in size and was p53 positive (100%), 57 tumours were >1 cm and <2 cm (18 p53 positive), 65 were >2 cm and <3 cm (30 p53 positive), 19 were >3 cm and <4 cm (11 p53 positive), and 4 were <5 cm in size (1 p53 positive).

Lymph node stage data was available on 137 patients. One hundred and one of the tumours were lymph node Stage A (axillary lymph node negative), 20 were Stage B (low axillary lymph node tumour positive) and 16 Stage C (operable high axillary lymph node positive). Of the Stage A tumours, 41 (40.6%) of cases were p53 positive, of the Stage B tumours eight (40.0%) of cases were p53 positive, and of the Stage C tumours seven (43.7%) of the cases were p53 positive.

The primary tumours of 17 of 31 (54.8%) patients with distant tumour metastases showed p53 protein expression as compared to 44 (38.3%) of the p53 positive primary tumours in the subgroup of 115 patients without evidence of metastatic disease. Twenty-six local recurrences of tumour were identified and of these the primary tumour was p53 positive in 12 cases (46.1%).

### Statistical analysis

### *p53, EGFR, c-*erb*B-2, and oestrogen receptor*

In an analysis of the 146 cases of invasive carcinoma examined, a statistically significant association of p53 protein expression and high tumour grade was found, $\chi^2 = 14.1$ (d.f. = 2), $P < 0.001$ (see Table II). Using chi-square analysis a highly statistically significant association of lack of tumour p53 protein expression and positive tumour oestrogen receptor protein status was identified, $\chi^2 = 19.78$ (d.f. = 1), $P < 0.001$ (see Table III). An association of p53 protein expression and tumour EGFR expression; $\chi^2 = 7.07$ (d.f. = 1), $P < 0.01$ and positive tumour c-*erb*B-2 status was also seen $\chi^2 = 4.61$ (d.f. = 1), $P = 0.032$ (see Table III).

### *Clinicopathological associations and patient survival*

There was a trend towards significance of p53 expression and increased regional tumour recurrence in 143 patients on whom recurrence data was available $\chi^2 = 3.20$ (d.f. = 1),

**Table II** Tumour p53, EGFR and c-*erb*B-2 immunoreactivity and tumour grade

| *Tumour Grade* | *p53 POS/NEG* | *EGFR POS/NEG* | *c-*erb*B-2 POS/NEG* |
|---|---|---|---|
| 1 | 3/18 | 5/16 | 3/18 |
| 2 | 15/32 | 12/35 | 20/27 |
| 3 | 43/35 | 31/47 | 35/43 |
| | $P < 0.001$ (S) | $P = 0.16$ (NS) | $P = 0.035$ (S)[a] |

[a](S) Statistically significant; (NS) Not statistically significant.

**Table III** Tumour p53, EGFR, c-*erb*B-2 staining and oestrogen receptor status

| | *p53 Protein expression* | |
|---|---|---|
| | *POS* | *NEG* |
| EGFR pos | 28 | 20 |
| EGFR neg | 33 | 65 |
| $\chi^2 = 7.07$ (d.f. = 1); $P<0.01$ (S)[a] | | |
| c-*erb*B-2 pos | 31 | 27 |
| c-*erb*B-2 neg | 30 | 58 |
| $\chi^2 = 4.61$ (d.f. = 1); $P = 0.032$ (S)[a] | | |
| Oestrogen receptor pos | 26 | 67 |
| Oestrogen receptor neg | 29 | 12 |
| $\chi^2 = 19.78$ (d.f. = 1); $P<0.001$ (S)[a] | | |

[a](S) statistically significant.

$P = 0.074$. There was no association between p53 protein expression and primary tumour size $\chi^2 = 6.52$ (d.f. = 4), $P = 0.164$, or lymph node stage (Stage A – lymph node negative, Stage B and C – operable lymph node positive) $\chi^2 = 0.065$ (d.f. = 2), $P = 0.97$ in the 137 patients on whom lymph node stage data was available. There was also no association between p53 protein expression and presence of distant metastases $\chi^2 = 2.12$ (d.f. = 1), $P = 0.145$, or local tumour recurrence $\chi^2 = 0.067$ (d.f. = 1), $P = 0.795$.

A non-significant trend towards significance of p53 expression and poorer patient survival was identified in the subgroup of 61 patients with tumours showing p53 expression; $\chi^2 = 3.76$ (d.f. = 1), $P = 0.053$ (see Figure 2).

### Discussion

In this study of 149 cases of primary symptomatic breast carcinoma taken from the Nottingham/Tenovus Primary Breast Carcinoma Series (146 invasive carcinomas and three cases of DCIS) we have demonstrated a statistically significant association between expression of the p53 protein and high tumour grade, expression of EGFR, and c-*erb*B-2 protein overexpression. A strong negative association of p53 protein expression and positive tumour oestrogen receptor status was also identified. A non-statistically significant trend towards poorer survival was identified in the subgroup of 62 cases (42.5%) that showed p53 expression (see Figure 2). Other groups have also found a similar association of p53 protein expression in breast carcinoma and high tumour grade (poor tumour differentiation) (Cattoretti *et al.*, 1988*a*; Ostrowski *et al.*, 1991; Walker *et al.*, 1991) with a similar trend towards poorer survival in patients with tumours expressing the p53 protein noted in one other published series (Ostrowski *et al.*, 1991).

A statistically significant association of p53 and c-*erb*B-2 overexpression was identified. This association has not been previously reported. Three other previous studies failed to find an association between p53 protein expression and c-*erb*B-2 overexpression (Davidoff *et al.*, 1991*b*; Horak *et al.*, 1991; Walker *et al.*, 1991). A significant association between p53 and EGFR expression has been noted in two other published series (Cattoretti *et al.*, 1988; Horak *et al.*, 1991). Other authors have also reported a similar relationship of p53 protein expression and negative tumour oestrogen receptor status in breast carcinoma (Cattoretti *et al.*, 1988*a*,*b*; Davidoff *et al.*, 1991*b*; Walker *et al.*, 1991) although Horak *et al.* were unable to confirm this finding (Horak *et al.*, 1991). The lack of association of p53 protein expression and primary breast tumour size has not been previously reported to our knowledge.

From our data p53 protein expression does not appear to be a specific feature of subtypes of invasive mammary carcinoma although special types of carcinoma were not present in sufficient numbers to draw definite conclusions about expression of p53 in rarer types. Expression of p53 protein does however appear to be rare in invasive lobular carcinomas of classical type. This study has confirmed earlier findings in other studies showing a relationship between p53 protein expression and high tumour grade, EGFR expression and a negative association with oestrogen receptor status. A non-significant survival disadvantage was seen in patients with tumours showing p53 protein expression as was also shown in one other previous study. We have also identified a relationship between p53 protein expression and immunocytochemical overexpression of the c-*erb*B-2 protein, a finding which was not identified in three other published series. Other published studies have not examined the relationship between p53 and local tumour recurrence, regional recurrence, or presence of distant metastases. We have also shown that expression of p53 protein is less common in the special types of breast carcinoma such as tubular, tubular/mixed, or lobular which tend to be of lower histological grade, although insufficient numbers of special types of mammary carcinoma were available for meaningful subgroup analysis by tumour type.

The results of immunohistochemical analysis of the p53 gene protein in both our own and in other series have shown that p53 protein expression in neoplastic cells is usually nuclear, although some tumour cell cytoplasmic staining may also be identifiable (Bartek *et al.*, 1991; Cattoretti *et al.*, 1988; Iggo *et al.*, 1990). Tumour cell p53 staining appears to be associated with p53 gene mutation, at least in some cases (Davidoff *et al.*, 1991*a*; Iggo *et al.*, 1990), as well as in breast carcinoma cell lines (Bartek *et al.*, 1990*b*), although altered

**Figure 2** Survival of patients by primary tumour p53 protein expression. The subgroup of patients with tumours showing p53 expression just fails to achieve statistical significance.

p53 protein expression may also be related to altered and abnormal p53 protein degradation in certain situations (Wynford-Thomas, 1992).

The presence of p53 protein expression in some cases of pure DCIS (one of three in our series) was also noted in three other series (Bartek *et al.*, 1990*a*; Davidoff *et al.*, 1991*a*; Walker *et al.*, 1991). This finding supports the hypothesis that p53 protein expression and by inference p53 gene mutation is present in the early stages of human breast cancer. Davidoff *et al.* showed that two of 15 cases of pure DCIS in their series expressed high levels of p53 protein. Analysis of the p53 mRNA from one of these two cases by polymerase chain reaction demonstrated a p53 mRNA nucleotide substitution, infering altered the amino-acid composition of p53 protein (Davidoff *et al.*, 1991*a*). This implies that p53 gene mutations may be present in the early stages of breast cancer. Expression of the same mutant p53 mRNA was also identified in both the *in situ* and invasive components of the same breast carcinoma, implying that p53 gene mutations may be maintained in the progression of breast carcinoma from DCIS to invasive disease.

If the hypothesis that p53 gene mutation is an early event in breast carcinoma is correct it is not surprising that there is no association between p53 protein expression and lymph node tumour status, distant metastases, or local recurrence of carcinoma. Other series have also observed a similar lack of association of p53 protein expression and lymph node status (Cattoretti *et al.*, 1988; Ostrowski *et al.*, 1991; Walker *et al.*, 1991) although an association of p53 expression and advanced lymph node status was reported by Davidoff *et al.* (1991*b*). Our data showing a lack of association of p53 protein expression, presence of distant metastases and local tumour recurrence would also be consistent with our finding of a lack of association of p53 protein expression and breast primary tumour lymph nodal status as described above.

p53 gene mutations are relatively common in invasive breast carcinoma (Prosser *et al.*, 1990; Thompson *et al.*, 1990) and have also been identified in breast carcinoma cell lines (Bartek *et al.*, 1990*b*). The wild type p53 gene has been shown to act as a suppressor of cellular growth in human breast carcinoma cells, as shown by a study of MDA-MB468 and T47D breast carcinoma cell lines which contain the mutant p53 gene, but which then subsequently fail to grow after DNA transfection with the wild type p53 gene (Casey *et al.*, 1991).

Recent evidence points to clonal expansion of mutant p53 gene containing cells in the progression of primary brain tumours (Sidransky *et al.*, 1992), implying that tumour cells which show p53 gene mutations may have a selective growth advantage as compared to tumour cells without p53 gene mutations, as predicted by Nowell (1976). Expression of the p53 gene protein appears to be a common event in human breast cancer and expression is directly related to poor tumour grade, epidermal growth factor receptor expression, c-*erb*B-2 protein overexpression, and negative tumour oestrogen receptor protein status.

We were unable to show that p53 protein expression is an independent prognostic factor for patient survival, although a near significant tendency towards poorer survival was seen in tumours that showed p53 protein expression. Our data would be consistent with p53 protein expression, and by inference p53 gene mutation or alteration in cellular p53 protein metabolism and degradation being an early event in human breast cancer. The recent observation that clonal expansion of mutant p53 containing tumour cell lineages occurs in primary cerebral gliomas would offer an attractive if incomplete explanation of our own findings if similar clonal expansion were also to occur in mammary carcinogenesis.

We are grateful to the Cancer Research Campaign and Trent Regional Health Authority for financial support, to Bill Brackenbury for photomicrography, and to Jinny Spence for typing the manuscript.

### References

BANKS, L., MATLASHEWSKI, G. & CRAWFORD, L. (1986). Isolation of human p53 specific monoclonal antibodies and their use in the studies of human p53 expression. *Eur. J. Biochem.*, **159**, 529–534.

BARTEK, J., BARTKOVA, J., VOJTESEK, B., STASKOVA, Z., REJTHAR, A., KOVARIK, J. & LANE, D.P. (1990*a*). Patterns of expression of the p53 tumour suppressor in human breast tissues and tumours *in situ* and *in vitro*. *Int. J. Cancer*, **46**, 839–844.

BARTEK, J., IGGO, R., GANNON, J. & LANE, D.P. (1990*b*). Genetic and immunochemical analysis of mutant p53 in human breast cancer cell lines. *Oncogene*, **5**, 893–899.

BARTEK, J., BARTKOVA, J., VOJTESEK, B., STASKOVA, Z., LUKAS, J., REJTHAR, A., KOVARIK, J., MIDGLEY, C.A., GANNON, J.V. & LANE, D.P. (1991). Aberrant expression of the p53 oncoprotein is a common feature of a wide spectrum of human malignancies. *Oncogene*, **6**, 1699–1703.

CASEY, G., LO-HSUEH, M., LOPEZ, M.E., VOGELSTEIN, B. & STANBRIDGE, E.J. (1991). Growth suppression of human breast cancer cells by the introduction of a wild-type p53 gene. *Oncogene*, **6**, 1791–1797.

CATTORETTI, G., RILKE, F., ANDREOLA, S., D'AMATO, L. & DELIA, D. (1988*a*). p53 expression in breast cancer. *Int. J. Cancer*, **41**, 178–183.

CATTORETTI, G., ANDREOLA, S., CLEMENTE, C., D'AMATO, L. & RILKE, F. (1988*b*). Vimentin and p53 expression on epidermal growth factor receptor positive, oestrogen receptor negative breast carcinomas. *Br. J. Cancer*, **57**, 353–357.

DANG, C.V. & LEE, W.M.F. (1989). Nuclear and nucleolar targeting sequences of c-erb-A, c-myb, N-myc, p53, HSP70, and HIV tat proteins. *J. Biol. Chem.*, **264**, 18019–18023.

DAVIDOFF, A.M., KERNS, B.J.M., IGLEHART, J.D. & MARKS, J.R. (1991*a*). Maintenance of p53 alterations throughout breast cancer progression. *Cancer Res.*, **51**, 2605–2610.

DAVIDOFF, A.M., HERNDON, J.E., GLOVER, N.S., KERNS, B.J.M., PENCE, J.C., IGLEHART, J.D. & MARKS, J.R. (1991*a*). Relation between p53 overexpression and established prognostic factors in breast cancer. *Surgery*, **110**, 259–264.

DONEHOWER, L.A., HARVEY, M., SLAGLE, B.L., MCARTHUR, M.J., MONTGOMERY, C.A., BUTEL, J.S. & BRADLEY, A. (1992). Mice deficient for p53 are developmentally normal but susceptible to spontaneous tumours. *Nature*, **356**, 215–221.

ELLIS, I.O., GALEA, M., BROUGHTON, N., LOCKER, A., BLAMEY, R.W. & ELSTON, C.W. (1992). Pathological prognostic factors in breast cancer II. Histological type. Relationship with survival in a large study with long term follow-up. *Histopathology*, **20**, 479–489.

ELSTON, C.W. & ELLIS, I.O. (1991). Pathological prognostic factors in breast cancer. I. The value of histological grade in breast cancer: experience from a large study with long-term follow-up. *Histopathology*, **19**, 403–410.

FINLAY, C.A., HINDS, P.W., TAN, T.H., ELIYAHU, D., OREN, M. & LEVINE, A.J. (1988). Activating mutations for transformation by p53 produced a gene product that forms an hsc70-p53 complex with an altered half-life. *Mol. Cell. Biol.*, **8**, 531–539.

HARRIS, A.L. (1991). Telling changes of base. *Nature*, **350**, 377–378.

HOLLSTEIN, M., SIDRANSKY, D., VOGELSTEIN, B. & HARRIS, C.C. (1991). p53 mutations in human cancers. *Science*, **253**, 49–53.

HORAK, E., SMITH, K., BROMLEY, L., LEJEUNE, S., GREENALL, M., LANE, D. & HARRIS, A.L. (1991). Mutant p53, EGF receptor and c-erbB-2 expression in human breast cancer. *Oncogene*, **6**, 2277–2284.

IGGO, R., GATTER, K., BARTEK, J., LANE, D. & HARRIS, A.L. (1990). Increased expression of mutant forms of p53 oncogene in primary lung cancer. *Lancet*, **335**, 675–679.

ISOBE, M., EMANUEL, B.S., GIVOL, D., OREN, M. & CROCE, C.M. (1986). Localization of gene for human p53 tumour antigen to band 17p13. *Nature*, **320**, 84–85.

LANE, D.P. & CRAWFORD, L.V. (1979). T-antigen is bound to a host protein in SV-40 transformed cells. *Nature*, **278**, 261–263.

LINZER, D.I.H. & LEVINE, A.J. (1979). Characterization of a 54K dalton cellular SV40 tumor antigen present in SV40-transformed cells and uninfected embryonal carcinoma cells. *Cell*, **17**, 43–52.

LOVEKIN, C., ELLIS, I.O., LOCKER, A., ROBERTSON, J.F.R., BELL, J., NICHOLSON, R., GULLICK, W.J., ELSTON, C.W. & BLAMEY, R.W. (1991). c-erbB-2 oncoprotein expression in primary and advanced breast cancer. *Br. J. Cancer*, **63**, 439-443.

MACKAY, J., STEEL, C.M., ELDER, P.A., FORREST, A.P.M. & EVANS, H.J. (1988). Allele loss on short arm of chromosome 17 in breast cancers. *Lancet*, **ii**, 1384-1385.

MELERO, J.A., TUR, S. & CARROLL, R.B. (1980). Host nuclear proteins expressed in simian virus 40-transformed and infected cells. *Proc. Natl Acad. Sci. USA*, **77**, 97-101.

MILNER, J. & WATSON, J.V. (1990). Addition of fresh medium induces cell cycle and conformational changes in p53, a tumour suppressor protein. *Oncogene*, **5**, 1683-1690.

MILNER, J. (1991). The role of p53 in the normal control of cell proliferation. *Curr. Opin. Cell. Biol.*, **3**, 282-286.

NOWELL, P.C. (1976). The clonal evolution of tumor cell populations. *Science*, **194**, 23-28.

OSTROWSKI, J.L., SAWAN, A., HENRY, L., WRIGHT, C., HENRY, J.A., HENNESSY, C., LENNARD, T.J.W., ANGUS, B. & HORNE, C.H.W. (1991). p53 expression in human breast cancer related to survival and prognostic factors: an immunohistochemical study. *J. Pathol.*, **164**, 75-81.

PROSSER, J., THOMPSON, A.M., CRANSTON, G. & EVANS, H.J. (1990). Evidence that p53 behaves as a tumour suppressor gene in sporadic breast tumours. *Oncogene*, **5**, 1573-1579.

RAYCROFT, L., WU, H. & LOZANO, G. (1991). Transcriptional activation by wild-type but not transforming mutants of the p53 anti-oncogene. *Science*, **249**, 1049-1051.

SAINSBURY, J.R.C., FARNDON, J.R., NEEDHAM, G.K., MALCOLM, A.J. & HARRIS, A.L. (1987). Epidermal growth factor receptor status as predictor of early recurrence of and death from breast carcinoma. *Lancet*, **i**, 1398-1402.

SIDRANSKY, D., MIKKELSEN, T., SCHWECHHEIMER, K., ROSENBLUM, M.L., CAVANEE, W. & VOGELSTEIN, B. (1992). Clonal expansion of p53 mutant cells is associated with brain tumour progression. *Nature*, **355**, 846-847.

SLAMON, D.J., GODOLPHIN, W., JONES, L.A., HOLT, J.A., WONG, S.G., KEITH, D.E., LEVIN, W.J., STUART, S.G., UDOVE, J., ULLRICH, A. & PRESS, M.F. (1989). Studies of HER-2/neu proto-oncogene in human breast and ovarian cancer. *Science*, **244**, 707-712.

THOMPSON, A.M., STEEL, C.M., CHETTY, U., HAWKINS, R.A., MILLER, W.R., CARTER, D.C., FORREST, A.P.M. & EVANS, H.J. (1990). p53 gene mRNA expression and chromosome 17p allele loss in breast cancer. *Br. J. Cancer*, **61**, 74-78.

TODD, J.H., DOWLE, C., WILLIAMS, M.R., ELSTON, C.W., ELLIS, I.O., HINTON, C.P., BLAMEY, R.W. & HAYBITTLE, J.L. (1987). Confirmation of a prognostic index in primary breast cancer. *Br. J. Cancer*, **56**, 489-492.

VENTER, D.J., TUZI, N.L., KUMAR, S. & GULLICK, W.J. (1987). Overexpression of the c-erbB-2 oncoprotein in human breast carcinomas: immunohistological assessment correlates with gene amplification. *Lancet*, **ii**, 69-72.

WALKER, R.A., DEARING, S.J., LANE, D.P. & VARLEY, J.M. (1991). Expression of p53 protein in infiltrating and *in-situ* breast carcinomas. *J. Pathol.*, **165**, 203-211.

WYNFORD-THOMAS, D. (1992). p53 in tumour pathology: can we trust immunocytochemistry. *J. Pathol.*, **166**, 329-330.